# Science Classroom Inquiry (SCI) Simulations: A Novel Method to Scaffold Science Learning

**DOI:** 10.1371/journal.pone.0120638

**Published:** 2015-03-18

**Authors:** Melanie E. Peffer, Matthew L. Beckler, Christian Schunn, Maggie Renken, Amanda Revak

**Affiliations:** 1 Department of Educational Psychology, Special Education and Communication Disorders, Georgia State University, Atlanta, Georgia, United States of America; 2 Conservation Education Department, Pittsburgh Zoo and PPG Aquarium, Pittsburgh, Pennsylvania, United States of America; 3 Wayne & Layne LLC, Minneapolis, Minnesota, United States of America; 4 Learning Research and Development Center, University of Pittsburgh, Pittsburgh, Pennsylvania, United States of America; University of Westminster, UNITED KINGDOM

## Abstract

Science education is progressively more focused on employing inquiry-based learning methods in the classroom and increasing scientific literacy among students. However, due to time and resource constraints, many classroom science activities and laboratory experiments focus on simple inquiry, with a step-by-step approach to reach predetermined outcomes. The science classroom inquiry (SCI) simulations were designed to give students real life, authentic science experiences within the confines of a typical classroom. The SCI simulations allow students to engage with a science problem in a meaningful, inquiry-based manner. Three discrete SCI simulations were created as website applications for use with middle school and high school students. For each simulation, students were tasked with solving a scientific problem through investigation and hypothesis testing. After completion of the simulation, 67% of students reported a change in how they perceived authentic science practices, specifically related to the complex and dynamic nature of scientific research and how scientists approach problems. Moreover, 80% of the students who did not report a change in how they viewed the practice of science indicated that the simulation confirmed or strengthened their prior understanding. Additionally, we found a statistically significant positive correlation between students’ self-reported changes in understanding of authentic science practices and the degree to which each simulation benefitted learning. Since SCI simulations were effective in promoting both student learning and student understanding of authentic science practices with both middle and high school students, we propose that SCI simulations are a valuable and versatile technology that can be used to educate and inspire a wide range of science students on the real-world complexities inherent in scientific study.

## Introduction

Recent calls related to science education highlight both the importance of inquiry and a pedagogical approach that presents science as a dynamic process that provides a unique way of “knowing” about the world in which we live [1]. The Next Generation Science Standards (NGSS) outline science practices as one of the three dimensions essential for creating student success in science education. These science practices refer to the beliefs and skill sets scientists use in their discipline-specific inquiry [2]. Although inquiry is recognized as important for the development of scientific reasoning skills, many classroom activities in K12 and undergraduate settings are limited to simple, non-authentic inquiry experiments. Authentic science inquiry refers to activities that model the processes used by practicing scientists. Chinn and Malhotra [3] outline 11 features that make an educational activity authentic. They determined that inquiry activities found in textbooks (a commonly utilized educational tool) fail to model the cognitive processes associated with authentic science. They conclude that the types of simple inquiry that students are exposed to in K12 and undergraduate settings create an understanding of science that is inconsistent with actual scientific processes.

Creating effective and authentic science classroom experiences requires a consideration of authentic science practices. We use the term *authentic science practices* here to broadly refer to the features of authentic science inquiry proposed by Chinn and Malhotra [3] as well as a consideration of the students’ understanding of the deeper meaning of science including their epistemic beliefs, the process of scientific inquiry, and understanding of the nature of science (NOS). Epistemic beliefs are a situated set of beliefs related to a learner’s understanding about what it means to *know* and the nature of knowledge [4]. Scientific epistemic beliefs contain four different dimensions: the nature of knowledge as fixed or changing; the nature of knowledge as absolute or relative; the source of knowledge and if it can be challenged; and that the derivation of knowledge can be derived from known facts or engagement in critical thinking [4]. NOS beliefs center on the values and understanding associated with scientific knowledge [5]. NOS knowledge encompasses seven elements including: the dynamic nature of science; the role of creativity in science; the influence of widely held, or accepted beliefs on science; the empirical basis of science; the influence of culture on science; the differences between theories and laws in science; and the nature of observations in science [5]. NOS and science inquiry, although related, are often incorrectly used as synonymous terms [6]. Science inquiry refers to the processes utilized by scientists that lead to generation of new scientific knowledge. This is in contrast to NOS, which refers to philosophical underpinnings that distinguish the practice of science from other disciplines [6]. This distinction is reflected in the NGSS, which emphasize teaching scientific practices as separate from NOS. Furthermore, the NGSS stipulate that consideration of science practices in addition to content and crosscutting examples are essential to student success in the science classroom [2]. Therefore, consideration of authentic science practices when designing curricula is essential to creating educational experiences leading not only to improvement of students’ content knowledge, but also towards development of cognitive processes more representative of practicing scientists [7, 8].

One of the barriers to effectively modeling authentic science practices within the classroom is feasibility. Schools are limited by time, money, and safety concerns and consequently laboratory experiments are typically dispatched in a recipe-like format where students passively follow a set of instructions to effectively and safely reach an expected scientific outcome. Furthermore, various features of authentic science practices are often abstract and difficult for students to conceptualize therefore resulting in additional challenges for instructors. To teach authentic science practices adequately requires both teacher mastery of authentic science practices and utilization of a *cognitive apprenticeship model* in which the teacher makes his or her thought process visible to the students [9]. Formation of this cognitive apprenticeship will allow students to emulate the teacher’s example and consequently be better equipped for understanding abstract concepts. One of the essential parts of forming a cognitive apprenticeship is scaffolding. Scaffolding provides titrated support to guide students’ learning as they gain confidence and competency with a certain task.

The use of technology in the classroom offers new opportunities for teaching authentic science practices by allowing students to engage in inquiry activities that are otherwise not feasible with a typical classroom setting. In addition to giving both teachers and students more flexibility to perform scientifically authentic inquiry, simulations can also serve as scaffolds for helping students manage complicated tasks that are often inherent in authentic inquiry. Quintana et al [10] outline a strategic framework for successful use of scaffolding and give examples of how various technologies are used to scaffold students as they undertake inquiry tasks. The authors contend that the scaffolding provided through the use of simulations may make difficult inquiry tasks more accessible [10]. Overall, these technologies are promising candidates as educational tools to advance science education as they can simultaneously improve student understanding of concepts and processes while maintaining student motivation for learning [11].

The Science Classroom Inquiry Simulator (SCI-Sim) is a web-based technology that we developed to run SCI simulations. These SCI simulations aim to promote authentic science inquiry and teach authentic science practices in a safe, cost effective, and timely manner. We define simulations as computer-based, dynamic representations of an entity, potentially invisible, where the user can manipulate the parameters [11–13]. In the case of SCI, each simulation was designed to represent the dynamic, trial-and-error nature of authentic science inquiry and to be easily adaptable to any discipline or age level. As a scaffold, SCI simulations help students think critically about research questions and problems in a manner more reminiscent of real scientists. SCI simulations are not only a useful instructional tool, but have the potential to serve as a powerful assessment framework through the collection of real time student data. Instead of using pre/post measures, students input responses to various prompts and their rationale for responses as they work through the simulation in a virtual laboratory notebook.

One of the current gaps in research on technology-enabled learning is an understanding of the role simulations play in developing students’ authentic science practices. Furthermore, very little prior work has considered *students’* thoughts on simulations as instructional tools. Therefore, we field tested SCI simulations with five different groups of students ranging in age from 6–12^th^ grade enrolled in various extracurricular informal learning experiences. Our specific focus during field-testing was on students’ perceptions of the SCI simulations’ benefits. We found that students reported that their perceived level of learning and amount of thought needed to complete the simulations was high, although they found the tasks to be not overwhelmingly difficult. Importantly, students reported that using SCI simulations alone without any instruction on authentic science practices altered their understanding of the practices utilized by scientists. The change in understanding of authentic science practices was positively correlated with the amount of learning reported by students. Therefore, SCI simulations have the potential to grow into a powerful tool for teaching authentic science practices in a fun, student friendly, and cost effective manner.

## Materials and Methods

### SCI Simulation Engine

The SCI-Sim system is implemented as a website application to ensure cross-platform compatibility since various classrooms utilize a mixed-environment of personal computers running Windows, OSX, or GNU/Linux, plus smartphones and tablets. Therefore, any device with a web browser and networking capabilities can participate in a SCI simulation. The SCI-Sim architecture utilizes an abstraction layer to hide the details of website forms, databases, web servers, and even the HTML code of each page, allowing the simulation designer to instead focus on the scientific content and flow of the simulation. The SCI-Sim system can support multiple SCI simulations, and multiple users for each SCI simulation. The SCI-Sim engine was released as open-source software and published on the GitHub website: https://github.com/sci-sim/sci-sim.

**Fig 1:**
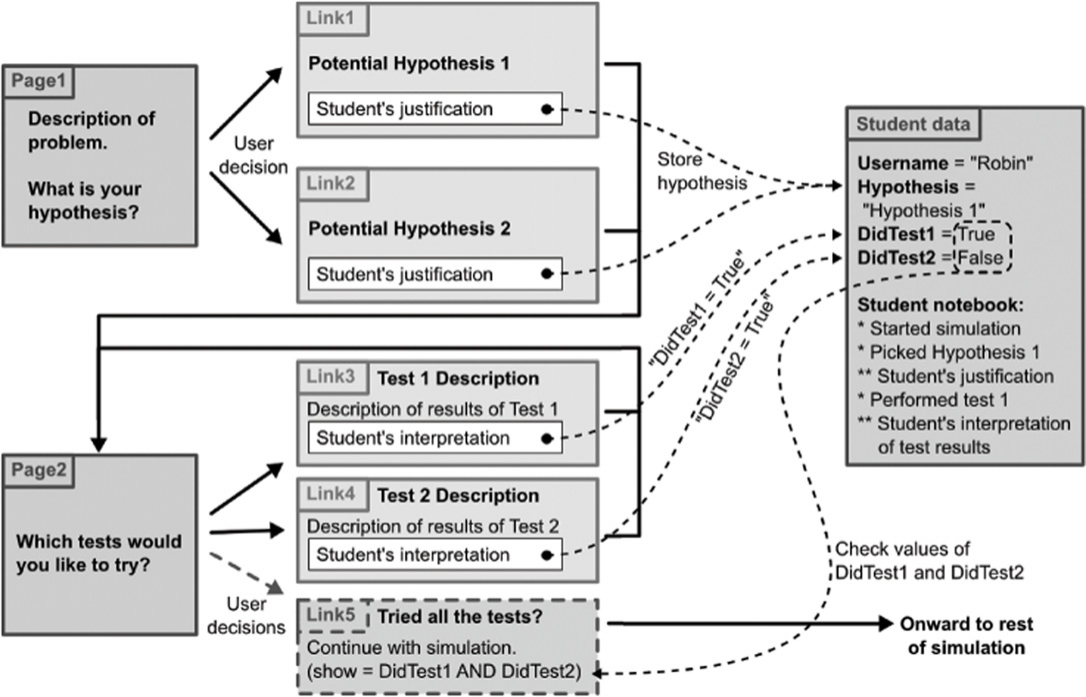
Schematic of SCI simulation architecture. After logging into the appropriate simulation, students read pertinent background information and are introduced to problem (i.e. unexplainable deaths of a certain animal species) and generate a hypothesis to explain the cause. Students are then asked to pick a hypothesis from a list of pre-generated hypotheses (*Seizing Sea Lions* and *Neural Tube Defects*) or to come up with their own hypothesis (*Unusual Mortality Events*) and then asked to give their rationale for their hypothesis. Students are then taken to the tests section of the simulation where they are given the option to try a variety of tests, in any manner of their choosing. After the student has completed a previously determined number of tests, a link appears (shown as Link 5 in the diagram) which allows the student to move forward in the simulation.

The scientific content of each SCI simulation is presented to the student as a series of interconnected pages. Although the simulation exists in a somewhat defined manner (introduction-hypothesis generation/revision/testing-conclusions) students are given autonomy as to exactly how they work through the simulation. Each page is designed to present the scientific content in an engaging multimodal fashion, integrating text, images, video, or any other technology. At the end of each page, one or more choices are presented, allowing the student to choose his or her path through the simulation (Fig. 1). As the student progresses through the simulation, every detail is recorded into a centralized database. These details include sample data points such as the time on each page and the path taken through the simulation, as well as more complex data, such as the students’ initial hypothesis and rationale, result interpretations, and conclusions. In addition to supplying a wealth of information for the instructor or researcher, the data recorded for each student enables the real-time, user-specific customization of the simulation. Each simulation is personalized by allowing student autonomy, specifically to pursue his or her hypotheses and chosen testing strategy. Furthermore, the simulation responds to inputs that each student makes. For example, the student's choice and justification of hypothesis can be used to customize the prompts of the simulation's conclusion, encouraging the student to reflect upon his or her original thought process and observe how it has changed given additional evidence.

Virtual lab notebooks offer additional personalization. Free-form entries may be made at any point in the simulation. Entries are also automatically added to the lab notebook when the student reaches important milestones in the simulation. For example, when he or she selects and justifies a hypothesis, the selection and justification are recorded in the lab notebook. The SCI-Sim also records which tests students perform along with the students’ interpretation of test results in the lab notebook. These record keeping features encourage use of cumulative evidence to support conclusions and offer a chance for student self-reflection on the process of investigation.

A central library feature is accessible from anywhere in the simulation, containing links to content the instructor thinks might be useful for the students. In the simulations highlighted here, these links included a variety of information such as general information about the species or people impacted by the target problem, news stories reporting on the issue, and general information that the student may or may interpret as relevant to the problem. For example, in the *Unusual Mortality Events* simulation, articles were included about the biology of manatees, dolphins, and brown pelicans, other examples of unusual mortality events, and reports of invasive species and harmful algal blooms in the Indian River Lagoon. The articles included in the library are selected to provide support if students choose to independently seek additional content information. Supplemental materials also give students ideas about potentially causal variable relevant to the targeted problem. To make the SCI simulations more authentic, the library does not point students towards one answer, but allows the student to evaluate a variety of probable causes and background information to aid in the development of an independent hypothesis.

For this study, three discreet SCI simulations were developed, each placing the student in the role of researcher tasked with solving a problem. The content for each simulation varied; two of the simulations were focused on historic and current unusual mortality events (*Seizing Sea Lions* and *Unusual Mortality Events*), and a third focused on a high rate of fetal neural tube defects occurring in a fictional town (*Neural Tube Defects)*. *Seizing Sea Lions* and *Unusual Mortality Events* were the most similar simulations, with the former focused on a historic unusual mortality event affecting sea lions in California [14] and the latter focused on the more recent unusual mortality events affecting brown pelicans, bottlenose dolphins, and manatees in the Indian River Lagoon and bottlenose dolphins along the eastern coast of the United States of America.

The simulation content was designed to be as accurate as possible based on the current scientific literature and discussion with scientists and conservation organizations. For example, in the *Unusual Mortality Events* simulation, the necropsy findings presented to students were based on the first authors’ personal correspondence with scientists associated with groups such as the Save the Manatee Club (Maitland, FL). In addition, the *Unusual Mortality Events* simulation included publicly available graphs generated by the National Oceanic and Atmospheric Administration and the St John’s River Water Management District. Therefore, to increase the authenticity of this experience, students were tasked with independently assessing and analyzing real world data about existing problems to formulate their own unique ideas.

SCI simulations take approximately one hour to complete, and although simulations follow a general template of introduction to a problem, hypothesis generation, hypothesis testing, and conclusions/future directions, students are given autonomy as to how they will complete the simulation. *Seizing Sea Lions* and *Neural Tube Defects* had a slightly different organization and incorporated additional information segments within the simulation such that after an initial round of hypothesis generation, testing, and conclusions students were introduced to the concept of domoic acid (*Seizing Sea Lions)* and folic acid (*Neural Tube Defects)*. Following introduction to additional concepts, students then entered into a second phase of hypothesis generation and testing.

*Unusual Mortality Events* differed from the other two simulations. For this simulation, instead of picking from a list of pre-defined hypotheses, students were required to generate their own hypothesis, perform up to 5 species-specific tests, generate a final conclusion and then extrapolate their findings to a different concurrent unusual mortality event. Students were also tasked with choosing one model species to study (manatees, dolphins, or pelicans) and justify their decision. *Unusual Mortality Events* also differed from the other two simulations since at the time of the study, there was no known unifying cause for these unusual mortality events. This is in contrast to *Seizing Sea Lions* and *Neural Tube Defects* as the root causes of each problem presented in these simulations were well established. Therefore, students using the *Unusual Mortality Events* could not easily find answers via the Internet or from prior knowledge, requiring a higher degree of creativity and problem solving on the part of the student.

### Participants

Participants in this study were students enrolled in various extracurricular informal educational activities in large mid-Atlantic city. As part of normal classroom activities, students worked through a SCI simulation. Since data used for this study was generated as part of normal informal classroom activities, the analysis of archived de-identified student data was determined by the Georgia State University Institutional Review Board (Protocol #H15146) to be not human subjects research and therefore waived the requirement for informed consent. Participation in this educational activity was voluntary and permission to collect student responses to these informal educational activities was provided informally. Student demographics include 29 middle school students and 59 high school students. Detailed information regarding gender and ethnicity was not collected, but participating students were of mixed ethnicities and gender. Additional information on each class setting can be found in Table 1.

**Table 1:** Description of five groups who utilized SCI simulations.

Group Number	Number of Students	Grade Level	Simulation	Program Description
1 (Setting 1)	26	8^th^-12^th^	“*Seizing Sea Lions*”	Extracurricular educational activity: Students met every other week for 6 meetings total, and one two-hour class session was devoted to using a SCI simulation.
2 (Setting 1)	17	8^th^-12^th^	“*Neural Tube Defects*”	Extracurricular educational activity: Students met daily for one week, and one two-hour class session was devoted to using a SCI simulation.
3 (Setting 1)	10	8^th^-12^th^	“*Unusual Mortality Events*”	Extracurricular educational activity: Students met weekly for 3 weeks. One two-hour class was devoted to using a SCI simulation.
4 (Setting 2)	14	11^th^-12^th^	“*Unusual Mortality Events*”	Pre-College Immersion Experience: Students met 2–3 times per week over the course of one month. One one-hour class was devoted to using a SCI simulation.
5 (Setting 3)	17	6^th^-8^th^	“*Unusual Mortality Events*”	Summer long research experience for middle school students: One two-hour class at the end of the program was devoted to using a SCI simulation.

### Design and Procedure

Students participated in one of three SCI simulations, *Seizing Sea Lions*, *Neural Tube Defects*, or *Unusual Mortality Events*. Prior to working through the simulation, students were given background information pertaining to the content of the simulation, but no information on authentic science practices. For example, prior to completing the *Seizing Sea Lions* simulation, students learned about regions of the brain and various brain imaging techniques. Students worked in groups of 2–4, depending on setting and computer availability, to complete SCI simulations.

Following completion of each SCI simulation, students individually completed anonymous paper-based surveys that we generated to obtain student feedback. Students were asked to rate on a 5-point Likert Scale the level of perceived difficulty, the amount of thought required, and how much the student felt like the simulation helped them to learn and understand the material. A response of one on our scale represented a perception of very little whereas a five indicating a great deal. Students were also asked *“Did this simulation change how you think about research and how scientists approach problems*? *Why or why not*?*”* to ascertain if their perception of authentic science practices changed as a result of completing a SCI simulation. Students responded to the question with a yes/no response and then gave open-ended justifications for their choice.

## Results

### General Student Perceptions of SCI Simulations

**Fig 2:**
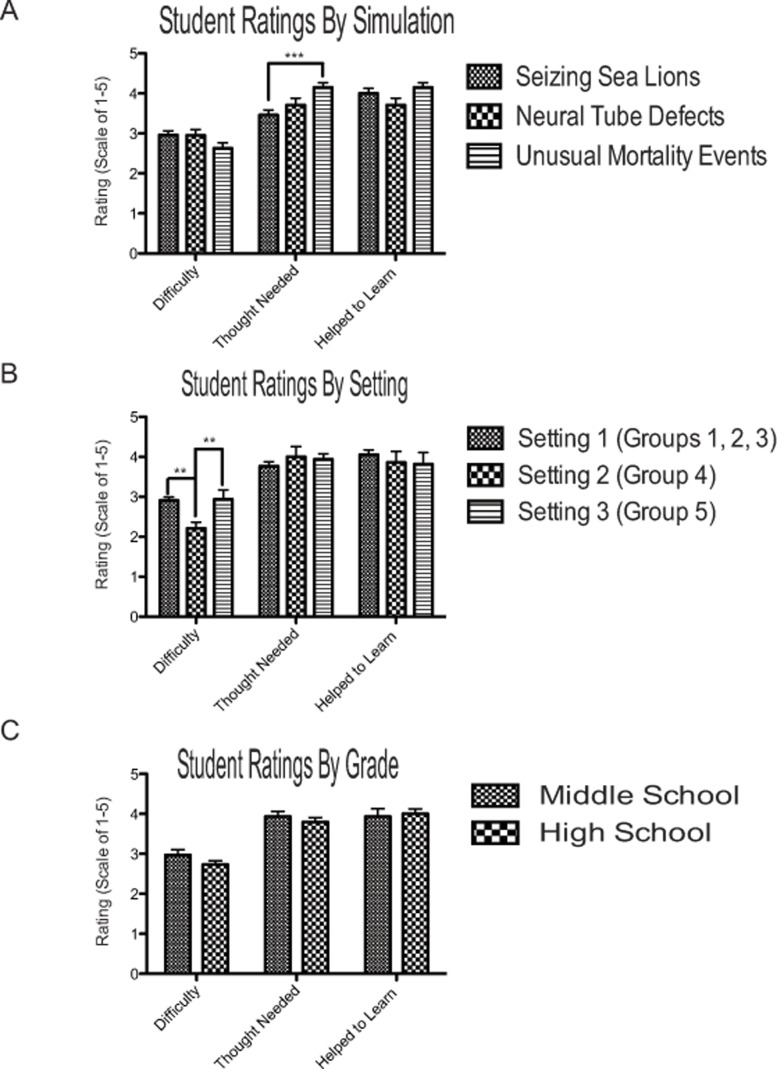
Student ratings of difficulty, thought needed, and learning efficacy are impacted by simulation and setting, but not grade. (A) No statistically significant differences were observed between simulations in regards to difficulty (One-way ANOVA, p > 0.05). However, students reported that the *Unusual Mortality Events* simulation required much greater thought to complete that the *Seizing Sea Lions* simulation (One-way ANOVA, *p* = 0.001). (B) The setting (Table 1) where the SCI simulations were used did not have an effect on the amount of thought needed to complete the simulation or how much the simulation helped the student to learn (One-way ANOVA, p > 0.05). However, the students in Setting 2 had an easier time completing the simulation than students in Setting 1 or 3 (One-way ANOVA, *p* = 0.004 and *p* = 0.014 respectively). (C) Analysis of middle school and high school students across all three settings indicated no statistically significant differences in difficulty, thought required to complete the simulation, or the amount the simulation helped them to learn (Independent samples t-test, *p* > 0.05). Graphs represent the average score on a 5-point Likert scale where 5 represents a high and 1 a low rating and error bars represent SEM.

To test whether students’ perceptions of simulation difficulty varied across simulations, we analyzed Likert Scale items using a one-way ANOVA. No statistically significant difference in students’ perception of simulation difficulty was identified across the three simulations, with an average rating of moderate difficulty, (mean = 2.81, SD = 0.741; Fig. 2A). *Seizing Sea Lions* and *Neural Tube Defects* had nearly identical ratings of perceived difficulty (mean = 2.96, SD = 0.528 and mean = 2.95, SD = 0.669, respectively). Notably, students reported that the *Unusual Mortality Events* simulation, the only SCI simulation in which students were required to generate their own hypothesis rather than choose from a pre-generated list, may have been slightly easier (mean = 2.63, SD = 0.859). The *Unusual Mortality Events* simulation was the most widely used simulation (Table 1) and this decreased level of perceived difficulty may stem from the fact that students in Setting 2 had a significantly easier time completing this particular simulation than in other settings (Fig. 2B).

Students also considered all simulations to be helpful for learning (mean 3.98, SD = 0.982). Analysis using a One-way ANOVA indicated no statistically significant differences in one simulation as more helpful than the others for the purposes of learning. *Seizing Sea Lions* and *Unusual Mortality Events* had very similar profiles (mean = 4.00, SD = 0.938 and mean = 4.07, SD = 1.058 respectively) although the *Neural Tube Defects* simulation was perceived as slightly less helpful (mean = 3.76, SD = 0.889).

Students also perceived that all three simulations required significant thought in order to complete (mean = 3.84, SD = 0.786). Using one-way ANOVA analysis, we identified a statistically significant difference in amount of thought required between *Seizing Sea Lions* (mean = 3.46, SD = 0.647) and *Unusual Mortality Events* (mean = 4.15, SD = 0.760), *p*<001. This greater perceived level of thought reported for *Unusual Mortality Events* may be due to the students being required to select their own species of focus, generate their own hypotheses, and extrapolate their findings to a concurrent, but geographically different unusual mortality event. Neither the *Seizing Sea Lions* nor *Unusual Mortality Events* simulations were perceived as requiring more or less thought than *Neural Tube Defects* (mean = 3.71, SD = 0.784).

We also compared student ratings across the three programmatic settings where SCI simulations were utilized. For the purposes of statistical analysis, Setting 1 is comprised of groups 1–3 (Table 1), as these were students enrolled in different courses offered within the framework of a single educational program. Settings 2 and 3 were distinct programs from Setting 1 (Table 1). Although there were no differences in the amount of thought needed or student perceived learning between settings, students in Setting 2 had a significantly easier time completing the simulation compared to students in both Setting 1 and Setting 3 (One-way ANOVA, p = 0.004 and p = 0.014 respectively, Fig. 2B). This is not surprising given that the students in Setting 2 were rising high school junior and seniors enrolled in a pre-college immersive experience and most likely had more experience with similar instructional activities.

**Fig 3:**
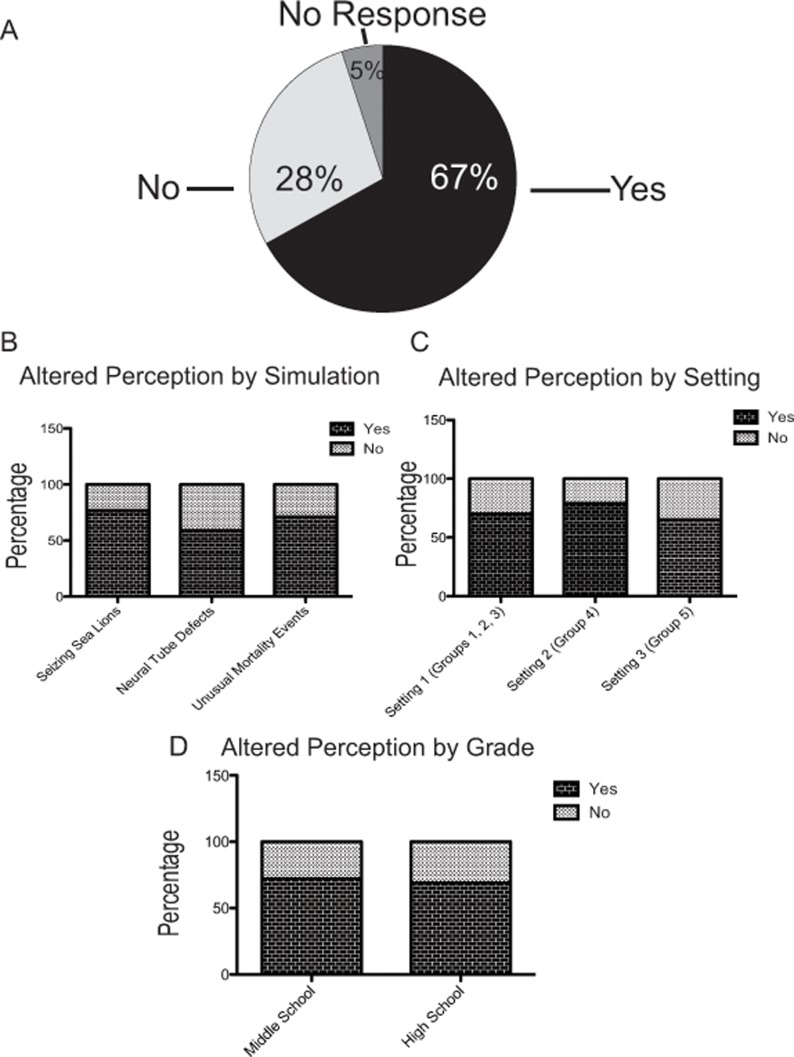
SCI simulations are effective at changing student’s perceptions of authentic science practices. (A) Of the 88 students who used the SCI simulations, 67% reported that use of the simulation altered their perceptions of the nature of authentic science practices. 28% of students indicated no change in perception of authentic science practices and 5% of students did not respond and were excluded from additional analyses. (B) All simulations were successful at changing students’ perceptions of authentic science practices, although no single simulation was more successful in this regard. (C) Regardless of setting, students reported changes in their perceptions of authentic science practices. (D) Grade level had no impact on whether students’ perceptions of authentic science practices changed, and students of both groups had a higher percentage of yes than no responses.

Among the 88 students who completed SCI simulations, 29 were in middle school and 59 were in high school. We observed no statistically significant differences between middle school and high school students among perceived difficulty, amount of learning, or amount of thought required (t-test, mean difficulty for middle school = 2.97, SE = 0.136 versus mean = 2.73, SE = 0.096 for high school; mean amount of learning for middle school = 3.93, SE = 0.198 versus mean = 4.00, SE = 0.123 for high school; mean thought required for middle school = 3.93, SE = 0.131 versus mean = 3.8, SE = 0.108 for high school; Fig. 3C). We also observed a slight trend indicating the middle school students perceived a higher degree of difficulty and required more thought to finish the simulation (Fig. 3C). Since our data set only included 88 students total, this trend may become more pronounced using a greater sample size.

### Changes in Student Perceptions of Authentic Science Practices

When asked if the simulation changed how they think about research and how scientists approach problems, 67% of students reported “yes,” while only 28% reported “no” (Fig. 3A). Students’ open-ended explanations noted that they were surprised to realize the complexity and difficulty inherent in the scientific process. Students gave the follow explanations:

“Yes, [the simulation] showed me the wide spectrum that scientists must consider when searching for the root of a problem”

“Yes, because I didn't know that [scientists] had so many steps to figure out what causes an animal to die out”

“Yes, because [scientists] need to take into consideration a lot of factors that do not seem like they could affect any results, but can”

“Yes, it takes quite a bit of research and understanding before any type of conclusion (if any) can be made about species and populations”

“Yes, it is a lot more work than I imagined. I had to make multiple hypotheses and make sure I can support them with evidence”

“Yes, because it is a lot more in depth and more complicated than I thought”

Notably, of the students who reported “no,” 80% indicated that their thinking had not changed, because the SCI simulation confirmed a prior belief about authentic science practices, for example stating:

“Not really, we have done projects like this at school”

“This simulation directly relates to what I learned in AP Bio [Advanced Placement Biology]. Your research might be completely wrong and you have to try a whole new perspective”

“No, I had rough thoughts before, but this simulation only confirmed my initial thoughts”

“No, I have had significant experience in the past with how scientists approach problems. The simulation strengthened it not changed it”

We used a Chi-Square analysis to determine the degree to which students changed their thoughts was related to the simulation they used, the setting they were in, or their age. We detected no effect of simulation (Fig. 3B, Pearson Chi-Square = 1.62, *df* = 2) or the degree to which the students changed their thoughts. Although not significant, students who worked through the *Seizing Sea Lions* and *Unusual Mortality Events* simulations tended to have higher percentages of “yes” responses (77% and 71% respectively) when compared to the students who used the *Neural Tube Defects* simulation (59%). Therefore, *Seizing Sea Lions* and *Unusual Mortality Events* simulation may be slightly more effective at changing students’ perceived understanding of authentic science practices.

We then assessed if setting had an impact on whether or not students reported that their understanding of authentic science practices had changed as a result of using a SCI simulation. We detected no statistically significant differences by setting (Pearson’s Chi Square = 0.72, *df* = 2. Interestingly, Setting 2 students reported the least amount of difficulty in completing the simulation, but the highest percentage of altered perceptions about authentic science practices (78% versus 70% in Setting 1 and 65% in Setting 3). Furthermore, using a T-test we observed no difference in reported changed perceptions of authentic science practices between middle and high school students (72% for middle school and 69% for high school), Fig. 3D. Note that the patterns we observe by simulation, setting, and grade level are different, ruling out any potential confounding factors among those three elements in producing the observed patterns.

Finally, we assessed whether setting, grade, simulation, difficulty reported, learning, or amount of thought needed to complete the simulation were correlated with the degree to which the students reported changing their thinking about authentic science practices. The only statistically significant correlation we observed was the relationship between changed perceptions of authentic science practices and how much the students reported the simulation helping them to learn. Interestingly, these two items were positively correlated (Table 2), suggesting that the overall use of the SCI simulations helped the students learn the subject material and also was effective at changing the students’ perceptions of authentic science practices.

**Table 2:** SCI simulations were successful at helping students gain content knowledge while promoting a more sophisticated understanding of authentic science practices.

Changed Thought X	R	*p*-value
Setting	-0.023	0.835
Grade	-0.035	0.755
Simulation	-0.046	0.68
Difficulty	-0.012	0.906
Thought Needed	0.105	0.344
Helped to Learn	0.32**	0.003**

## Discussion

The use of technology in the classroom is a powerful tool not only for supporting student learning, but also for allowing inquiries that are analogous to authentic science practice. Thus, SCI simulations have the potential to enrich students’ learning about the content presented, while at the same time scaffolding authentic science inquiry and helping students better understand authentic science practices. Overall, students reported that although simulations required a significant amount of thought to complete, they were perceived as only moderately difficult. Importantly, regardless of grade, setting, or simulation, students reported that their perceptions of authentic science practices changed without direct, explicit instruction. The degree to which students reported changes in the way they thought about science was also positively correlated with the level of students’ reported learning. We found that SCI simulations were appropriate for both middle and high school audiences. Although we detected no difference between middle school and high school students among any of the variables assessed, this study lacked sufficient power to do detailed analysis between students of different ages. Consequently, future work with a greater number of participating students may indicate possible developmental differences in student understanding of various facets of authentic science practices.

Previous research findings have indicated that participation in inquiry activities alone is insufficient to cause change in students’ understanding of authentic science practices, particularly those that relate to knowledge of NOS [5, 15, 16]. Sandoval [16, 17] suggests that the reason for this may be due to a lack of understanding of the students’ science epistemologies in inquiry activities. As used by [16], *scientific epistemology* is a subset of NOS that focuses on the students’ personal conceptions of the nature of *science* knowledge. Sandoval [16] suggests that analysis of students’ practical epistemologies (i.e., how *they* approach inquiry) and how these beliefs evolve to a more formal epistemology (i.e., how a *scientist* approaches inquiry) is essential for successful science education. By understanding student epistemologies and their development, we can better understand how to develop effective inquiry tasks, particularly with regard to teaching an understanding of authentic science practice. In the meantime, our initial analysis suggests that SCI is altering students’ perceptions of authentic science practices, potentially influencing the development of students’ formal epistemologies. Furthermore, work by [16] also suggests that paying attention to student practices (for example, how they approach the research questions presented in SCI) will yield insight into understanding students’ epistemological beliefs. This is particularly interesting and relevant to current gaps in research on the learning that occurs in simulations [11] because using a tool like SCI gives insights into the situated epistemology adolescents adopt in simulated, authentic science inquiry activities.

Students are prompted by the SCI-sim engine to reflect, in real time, on their choices. This automatic tracking gives a real-time insight into the students’ practical epistemology. As students progress through a SCI simulation, they are frequently prompted to describe their rationale for choosing a particular hypothesis, testing strategy and conclusions after discovering an important piece of data. These justifications, along with the path students take through the simulation and recorded field notes, provide a rich data source yielding insight into students’ thought processes. We expect this data source will be useful for both future student assessment and researcher study. For example, there is a need for effective assessments of students’ epistemological understanding within the context of authentic inquiry settings [19]. Importantly, these student process data are gathered in real time, allowing the investigator or teacher to collect student data without waiting until the end of an activity. By collecting data during the activity, a SCI simulation generates a more accurate picture of the students’ learning than what may be gleaned from a post-hoc activity. Furthermore, since the prompts are embedded within the simulation, there is no need to use a potentially distracting method that may influence students’ responses. As an educational tool, teachers can use SCI simulations to assess the most difficult parts of the task and the presence of common misconceptions.

To understand how the individual learner works through SCI simulations, future work will include post-simulation interviews querying students about their experimental choices. These reflective questions combined with the students’ choices will yield further insights into the students’ practical epistemologies. In addition, our data suggests that the authentic inquiry experience provided through SCI simulations is sufficient to change student perceptions of authentic science practices. Therefore, we propose pursuing more detailed analysis to ascertain if SCI simulations alone, or as scaffolds embedded within established practices for teaching inquiry and NOS, better improve student understanding of authentic science practices. By improving our understanding of what features of this inquiry experience are most effective and their relationship to students’ scientific epistemologists, we can design more effective classroom inquiry experiences.

Our metric to assess if students’ understanding of authentic science practices was altered as a result of participating in a SCI simulation included a single free response item, and no post simulation interviews. Therefore, to expand our understanding of exactly what portions of authentic science practices were altered, and if they were indeed altered (instead of only perceived), future experiments will include clinical interviews using a model such as presented by [18] and additional detailed post-simulation assessment items on various aspects of authentic science practices. For example, previously validated assessment tools, such as the Views About Scientific Inquiry [6] and Views of Nature of Science Questionnaire (VNOS) [15] would aid in teasing out which aspects of authentic science practices may be impacted by student participation in SCI simulations. Furthermore, our current data set did not include any items that would give insights into whether SCI simulations alter students’ epistemic beliefs. We therefore propose using metrics such as those used by [4]. Results from these measures would further support the use of SCI simulations as a technology that goes beyond simply teaching the content to a transformative learning experience that improves the students’ understanding of authentic science practices. We expect, based on our findings and on prior work, that this research will help to generate additional insights into broader learning gains offered by SCI simulations. In the meantime, the work presented here serves as a foundation for SCI simulations as a valuable and feasible tool to teach both conceptual understanding and authentic science practices, especially within broader educational experiences or programs. The SCI simulations are inexpensive and easily implemented with the potential for transforming both science education and science education research.
